# Design and Characterization of New D–A Type Electrochromic Conjugated Copolymers Based on Indolo[3,2-b]Carbazole, Isoindigo and Thiophene Units

**DOI:** 10.3390/polym11101626

**Published:** 2019-10-08

**Authors:** Yuling Zhang, Shuang Chen, Yan Zhang, Hongmei Du, Jinsheng Zhao

**Affiliations:** 1 State Key Laboratory of Heavy Oil Processing, College of Chemical Engineering, China University of Petroleum (East China), QingDao 266580, China; 18866264190@163.com; 2 Shandong Key Laboratory of Chemical Energy Storage and Novel Cell Technology, Liaocheng University, Liaocheng 252059, China; zy@lcu.edu.cn (Y.Z.); duhongmei@lcu.edu.cn (H.D.); 3 College of Chemistry and Chemical Engineering, Liaocheng University, Liaocheng 252059, China

**Keywords:** Indolo[3,2-b]carbazole, isoindigo, electrochromism, conjugated polymers, stille coupling

## Abstract

Two new donor–acceptor (D–A) type organic conjugated random copolymers were successfully synthesized by three-component Stille coupling polymerization of indolo[3,2-b]carbazole (ICZ), isoindigo (IID) and thiophene units, namely PITID-X (X = 1 and 2), with the controlled monomer feed ratios of 3:1:4 and 1:1:2, respectively. The strategy of incorporating different alkyl-branched donor/acceptor units and raw material feed ratios facilitated the improvement of optical properties, solubility, conjugated structure, and electrochromic performance. Cyclic voltammetry, UV-vis-NIR absorption spectra, kinetic and colorimetric measurements of the spray-coated films were recorded in the fabricated three-electrode cells. The results showed that PITID-2, whose optical/electrical properties were better than that of PITID-1, was the candidate electrochromic material due to low band gap of 1.58 eV accompanying the color changing from cyan (neutral state) to gray (oxidized state). The copolymer also illustrated fast bleaching/coloration response time of 2.04/0.33 and 1.35/1.50 s in a 4 s time interval, high coloration efficiency of 171.52 and 153.08 cm^2^ C^−1^ and stable optical contrast of 18% and 58% at the wavelength of 675 and 1600 nm, respectively.

## 1. Introduction

Π-conjugated organic polymers that have inherently remarkable electron or hole-transporting properties have been intensively investigated in optoelectronic materials for their huge potential application in various semiconductor devices, including electrochromic devices [1,2,3,4]. Comparing with inorganic electrochromic materials, organic conductive materials have the advantages of facile processing, convenient color control, and tunability in structure modification. The researchers have designed and synthesized many molecular units with donor–acceptor (D–A) structures using chemical or electrochemical polymerization. Following the D–A strategy, energy band-gap levels, structural characteristics, and electrochromic properties can be easily adjusted by introducing different monomer units and side chains [5,6]. The well-matched donor and acceptor units are widely used in low-band-gap copolymers, which contributes to the significant improvement of intramolecular charge transfer ability and the enhancement of conjugated backbone length with higher onset absorption wavelength and absorption intensity [7,8,9].

Among the versatile donors, acceptors, and their derivatives, the novel building blocks of indolo[3,2-b]carbazole (ICZ) and isoindigo (IIG) units have raised the researchers’ much attention [10,11]. ICZ is a well-performance electroactive organic chromophore commonly used as a donor due to its more rigid and extended conjugated structure than carbazole and indolo[3,2-b]indole units. [12,13]. Indolo[3,2-b]carbazole derivatives, whose 5,11-position can be substituted by long alkyl, aryl side chains, or other pendant units, contribute to modification of the π–π stacking and lamella structure on the stacked conjugated polymers [14,15,16]. The 2,8- substitution positions of ICZ monomer can be brominated or electrochemically polymerized. The corresponding derivatives have been explored intensively for their excellent photoelectronic performance in organic semiconductor devices [17]. Two redox-active diphenylamine blocks were introduced to the ICZ-based polymers. The obtained materials showed reversible near-infrared electrochromic and electroluminochromic properties with a high emission quantum yield of 30% [16]; Mula S et al. compared three kinds of ICZ-based complexes and the materials illustrated adjustable features and promising application in functional materials by density functional calculation [18]; Ethylbenzene, octyloxylbenzene, ethyloxylbenzene, and *N*,*N*-diethylaniline units were introduced to ICZ-based conjugate structure, the results illustrated wide absorption range and good light-harvesting ability in solar cells [19]. Our group and other researchers synthesized versatile ICZ derivatives to obtain functional conjugate structures and investigated their charge transporting, hole-transporting abilities and electrochromic properties in devices [20,21,22]. In addition, indolo[3,2-b]carbazole derivatives have been widely used as excellent organic lighting-emitting diodes (OLED) or organic field-effect transistors (OFEDs) due to their excellent hole-transporting properties. The novel rigid unit has drawn much attention as excellent photo- and electro- active or aggregation-induced fluorescence materials for adjustable the highest occupied molecular orbital/the lowest unoccupied molecular orbital (HOMO/LUMO) levels and energy band gap.

An isoindigo-based (IID) unit, a derivative of indigo, is an important organic conjugated candidate in conventional dye industry, as the material can be obtained from sustainable natural resources [23]. The nitrogen and oxygen atoms in the two lactam rings contribute to form intramolecular hydrogen bond and planar structure. This can enhance the effective conjugation length and π–π interaction leading to a narrow energy band gap and high extinction coefficiency [24,25]. The branched alkyl side chains of isoindigo-based derivatives have also been studied for electroluminescent devices and organic photovoltaic materials due to the advantages of its wide absorption range in the visible (380–730 nm) and near infrared region with large onset absorption wavelengths [26]. The strategy of introducing different side chains was applied to adjust the polymer solubility via the method of replacing the hydrogen atoms of the core unit. The properties of narrow band gap, broad absorption profiles can also be slightly adjusted. Romain et al. characterized a series of isoindigo-based copolymers to explore the properties of p-type and n-type charge transport in the thin films with thiophene-based blocks, the results illustrated potential application in electrochromic areas [27,28]. It is interesting to investigate the electrochromic properties of the copolymers consisting of indolo[3,2-b]carbazole, isoindigo and thiophene units.

We were motivated by the successive application of the D–A type organic conjugated polymer in organic optoelectronic areas, including electrochromism. The alternating donor and acceptor units could contribute to partial isolation of the frontier molecular orbitals. The donor unit contributes a lot to the HOMO levels, while the LUMO levels have more acceptor character [29]. Herein, we reported the synthesis and characterization of the two D–A type three-component copolymers based on ICZ, IID and thiophene units, namely PITID-1 and PIDIT-2, with long alkyl substituted side chains. The copolymers were synthesized via Stille cross-coupling polymerization method with high yields. ^1^H NMR, XPS and IR tests were conducted to confirm the structures of the two new random copolymers. The two solution-processable copolymers were spray-coated onto the conductive slide of indium tin oxide glasses (ITO), serving as working electrode. Additionally, the Ag wire and Pt wire were used as the pseudo-reference electrode and counter electrode, respectively. The cyclic voltammetry, absorption profiles, kinetic curves, and stability measurements were carried out to compare the corresponding electrochromic properties of the two copolymer films via UV-vis-NIR spectrophotometer, which was used in conjunction with electrochemical workstation.

## 2. Instrumentation and Materials

### 2.1. Instrumentation

^1^H NMR spectra of the two copolymers were recorded with deuterated chloroform (CDCl_3_, Cambridge Isotope Laboratories, Inc., Andover, USA) as solvent and tetramethylsilane (TMS) as internal standard with the equipment of Advance NEO 500 MHz spectrometer (Bruker BioSpin International AG, Switzerland). Cyclic voltammetry was carried out at the scan rate of 0.1 V/s from −2 V to 1.8 V with the CHI660D electrochemical workstation (Chen Hua Instrument Co., Ltd, Shanghai, China) in the self-fabricated three-electrode cell. The classical system used polymer-sprayed indium tin oxide (ITO, Zhuhai Kaivo Optoelectronic Technology Co., Ltd, Guangdong, China) substrate, Ag wire, and Pt wire serving as working electrode, pseudo-reference electrode, and counter electrode, respectively. A 0.2 M TBAPF_6_/ACN electrolyte solution was used during the electrochemical measurements, including cyclic voltammetry, kinetics, colorimetry and memory measurements. The redox peaks of ferrocenium/ferrocene (Fc^+^/Fc) solution were carried out to calibrate Ag wire to normalize HOMO/LUMO energy levels [30]. The UV-vis-NIR spectroelectrochemistry and kinetic spectra were recorded via Varian Cary 5000 spectrophotometer (Agilent Technologies Ltd, Mulgrave, Australia) which is used in conjunction with CHI660D electrochemical workstation by switching the stepwise voltages from neutral state to oxidized state. X-ray photoelectron spectroscopy (XPS) analyses of the copolymer films were measured on a thermo Escalab Xi^+^ (ThermoFisher Scientific, Waltham, USA) with a monochromated Al X-ray resource. Fourier transform infrared spectroscopy (FT-IR) was recorded in a Nicolet 6700 FTIR spectrometer (Thermo Nicolet Co., Wisconsin, USA), using potassium bromide as a carrier. Thermogravimetric analysis (TGA, TG, NETZSCH Scientific Instruments Trading Ltd., Germany) was measured to record the thermal stability of the two random copolymers at the heating rate of 10 °C /min from 20 to 750 °C. The pictures of spray-coated films at different redox states and as polymer solution were pictured by a canon camera. The information on molecular weight was obtained by gel permeation chromatography (GPC) analysis (Waters Co., Milford Massachusetts, USA), which was carried out on a waters 2414 system with a UV-detector. The test temperature was 80 °C, and the mobile phase was 1,2,4-trichlorobenzene, and polystyrene was used as the standard, a PSS SDV analytical column (5 μm, 300 mm × 8.0 mm, 10000 Å) was used for the measurement. Elemental analyses were performed with a PerkinElmer EA 2400 II elemental analyzer (Elementar Trading Co., Ltd, USA).

### 2.2. Materials

**Scheme 1:**
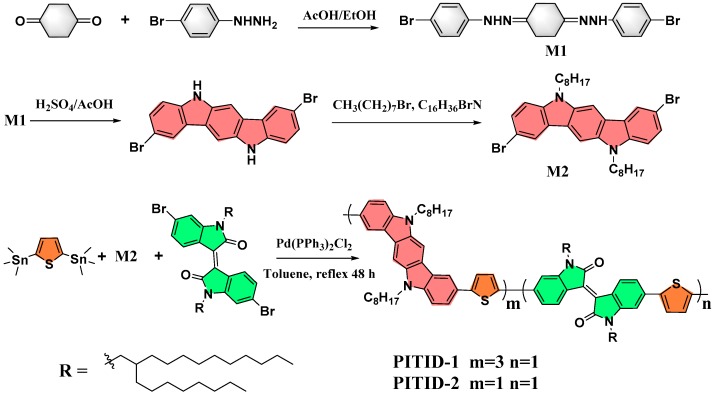
The synthetic route of the conjugated polymers PITID-1 and PITID-2.

All the reaction reagents and solvents were all purchased directly from commercial sources and used without further purification unless specifically noted. The monomer M1 and M2 were synthesized according to the reported synthetic procedures in the literatures with slightly modification [17,22]. The synthetic details and characterization data of the monomers M1, M2 have been reported previously in our group [22]. 2,5-bis(trimethylstannyl)thiophene and (E)-6,6’-dibromo-1,1’-bis(2-octyldodecyl)-[3,3’-biindolinylidene]-2,2’-dione were directly purchased from commercial resources (Derthon Optoelectronic Materials Science Technology Co., Ltd, Shenzhen, China) and used without further purification. Two isoindigo-based random copolymers PITID-X (X = 1 and 2) were synthesized by Stille cross-coupling polymerization with indolo[3,2-b]carbazole derivative and thiophene blocks in different raw material feed ratios of 1:3:4 and 1:1:2, respectively. The synthetic routes were shown in Scheme 1.

### 2.3. Synthesis of Monomers

The synthesis routes of the monomers M1 and M2 and the corresponding copolymers were presented in the Scheme 1. The intermediate monomers 2,8-dibromo-5,11-dihydrol-indolo[3,2-b] carbazole and 2,8-dibromo-5,11-dioctyl-indolo[3,2-b]carbazole were synthesized via the method of double fisher idolization according to the previous literature procedures [17,22].

### 2.4. Synthesis of Copolymer PITID-1

The monomers 2,5-bis(trimethylstannyl)thiophene (200 mg, 0.4881 mmol), (E)-6,6’-dibromo-1,1’-bis(2-octyldodecyl)-[3,3’-biindolinylidene]-2,2’-dione (119.72 mg, 0.1220 mmol), 2,8-dibromo-5,11-dioctyl-5,11-dihydroindolo[3,2-b]carbazole (233.8 mg, 0.3661 mmol), and the catalyst bis(triphenyl-phosphine)Palladium(II)Dichloride (Pd(PPh_3_)_2_Cl_2_) (20 mg, 0.0282 mmol, 5.78 mol %) were added into a round flask with 40 mL anhydrous toluene solution. The reaction apparatus were purged and re-filled with argon at least three times to remove excess air. The reaction mixture solution was stirred at 110 °C for 48 h, then cooled down to room temperature. The toluene solvent was removed under reduced pressure. The residues obtained after vacuum distillation of toluene solvent were re-dissolved in a small amount of chloroform, and then be precipitated by cold methanol. The crude product was obtained by filtration, and then subjected to the following purification steps by the soxhlet extraction method. In the soxhelt extraction procedure, the eluents selected in turn were n-hexane, anhydrous methanol, and acetone, successively. After drying under vacuum, the polymer as dark black solid was collected with a yield of 74%. ^1^H NMR (500 MHz, Chloroform-*d*) was given in Figure S1a (in Supplementary Materials), and the shifts of the hydrogen atoms and the assignments of their corresponding peaks are also given in the Figure S1a (in Supplementary Materials). A, Table 1 listed the molecular weight of the two polymers, including the number-average molecular (*M*_n_), the weight-average molecular (*M*_w_), and the polymer dispersity index (PDI) etc. The data for *M*_n_, *M*_w_, PDI of PITID-1 were 20.1, 27.5 kDa, and 1.37, respectively. Elemental analysis result: Anal. Calcd for PITID-1: C, 80.75; H, 8.72; S, 4.96; N, 4.33; Found: C, 80.48; H, 8.35; S, 5.02; N, 4.45.

### 2.5. Synthesis of Copolymer PITID-2

Compared with the synthesis produce of PITID-1, the reaction precursors 2,5-bis(trimethylstannyl)thiophene (300 mg, 0.7322 mmol), (E)-6,6’-dibromo-1,1’-bis(2-octyldodecyl)-[3,3’-biindolinylidene]-2,2’-dione (344.4 mg, 0.3661 mmol), and 2,8-dibromo-5,11-dioctyl-5,11-dihydroindolo[3,2-b]carbazole (234 mg, 0.3665 mmol), and the catalyst bis(triphenyl-phosphine)Palladium(II)Dichloride (Pd(PPh_3_)_2_Cl_2_, 30 mg, 0.04274 mmol, 5.84 mol %) were added into a round flask with 40 mL toluene solution. The synthesis and processing produres were the same as PITID-1. The dark black solid was collected with a yield of 73%. The ^1^H NMR spectrum of PITID-2 was shown in Figure S1b, and the shifts of the hydrogen atoms and the assignments of their corresponding peaks are also given in Figure S1b. Table 1 illustrated the data for *M*_n_, *M*_w_, and PDI, which were 17.7, 23.7 kDa, and 1.34, respectively. Elemental analysis result: Anal. Calcd for PITID-2: C, 80.27; H, 9.35; S, 4.37; N, 3.82. Found: C, 80.01; H, 8.91; S, 4.45, and N, 4.01.

## 3. Results and Discussions

### 3.1. FT-IR Spectra of the Two Polymers

**Figure 1:**
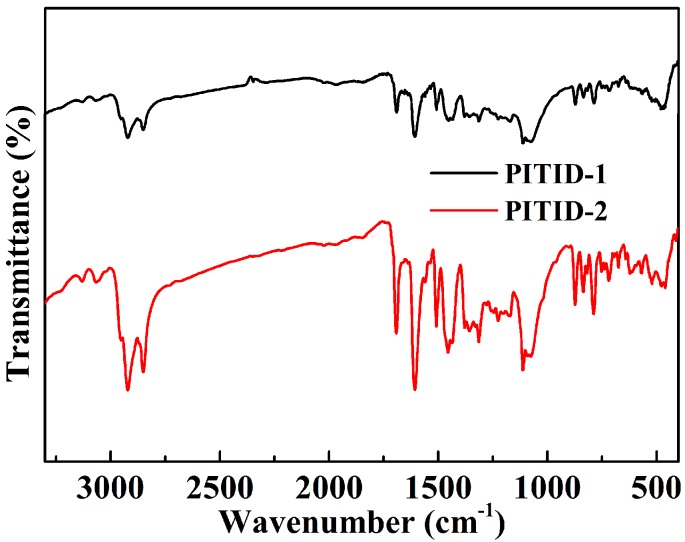
FTIR spectra of the two compounds PITID-1 (**black line**) and PITID-2 (**red line**).

To confirm the structures of the random copolymers with different donor/acceptor monomers, including thiophene, isoindigo, and indolo[3,2-b]carbazole derivative units, the FT-IR spectra of the two materials PITID-X (X = 1 and 2) were measured and illustrated in Figure 1. The transmittance curves showed that several similar characteristic peaks were identified due to the asymmetrical stretching vibration or the skeletal vibration of specific chemical bond/groups. For the polymer PITID-1, the peaks, including 2922 cm^−1^ (asymmetrical stretching vibration of –CH_2_– group), 2851 cm^−1^ (symmetrical stretching vibration of –CH_2_– group) were observed for the alkyl side chains in IIG or ICZ. The peak at 1690 cm^−1^ corresponded to the stretching vibration of ketone (C=O) in the isoindigo unit. Three peaks at 1607, 1508, 1452 cm^−1^ may derive from the skeletal vibration of the thiophene, ICZ, or IIG ring. The peak at 1313 cm^−1^ referred to the –C–N– stretching vibration of IIG or ICZ groups. The absorption peaks at 1170 cm^−1^, 1111 cm^−1^ were assigned to the in plane bending vibration of the C–H bonds alongside the skeleton of the whole polymer chain. Other peaks at 872, 787, and 718 cm^−1^ were due to the out-of-plane bending vibration of C–H in the aromatic rings. For FT-IR spectra of the PITID-2, the positions of characteristic peaks were similar to that of PITID-1, although the absorption intensity owed a slight difference. The results showed that the backbones of the two copolymers both contained the monomeric units mentioned above [31].

### 3.2. XPS Profiles of Polymer Films

**Figure 2:**
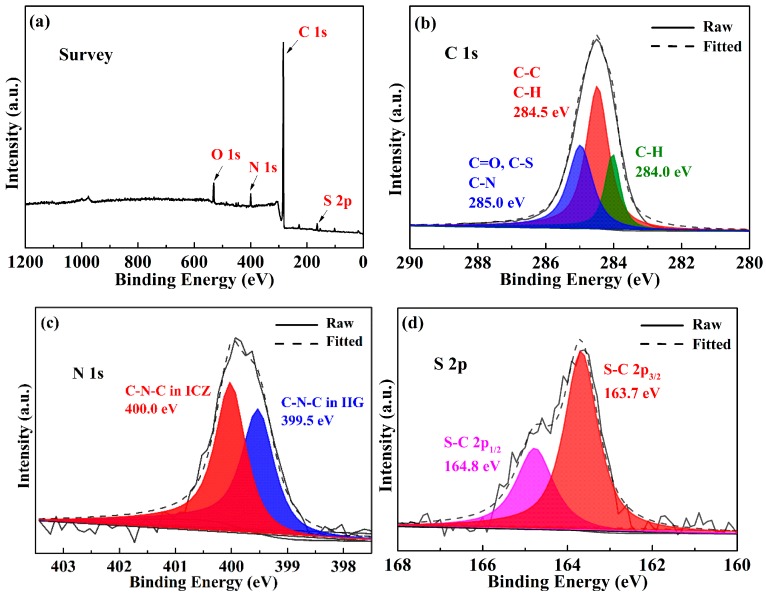

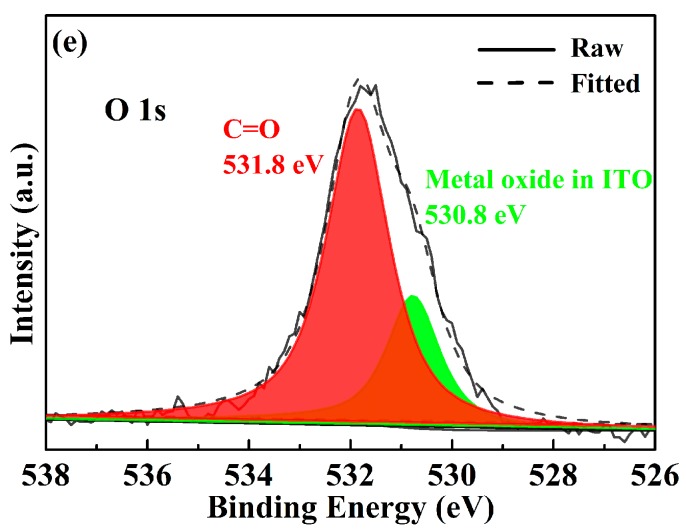
High-resolution X-ray photoelectron spectroscopy (XPS) spectra of the chemical-polymerized copolymer PITID-2 thin films: (**a**) Survey, (**b**) C 1s, (**c**) N 1s, (**d**) S 2p, (**e**) O 1s. The raw curve and the fitted curve were shown in solid and dotted lines, respectively.

In order to investigate the elemental compositions of carbon, nitrogen, oxygen, and sulphur atoms along the copolymer backbones, the materials PITID-1 and PITID-2 were characterized by X-ray photoelectron spectra (XPS) measurements. The results were recorded and shown in Figure 2 and Figure S2 (supporting information). Comparing the XPS profiles of the two copolymers, the two materials illustrated similar chemical state and bond energy intensity.

Taking the polymer PITID-2 for example, the survey scan revealed the presence of the four elements including carbon, nitrogen, sulphur, and oxygen, as shown in Figure 2a. The C 1s gross peak of the random copolymer PITID-2 can be deconvoluted into three peaks, as illustrated in Figure 2b. The peak located at a binding energy (B.E.) of 284.0 eV originated from C–H throughout the whole backbone. The peak located at 284.5 eV was attributed to C=C, which can be originated from the carbon atoms in the aromatic skeleton (C=C) of the monomers including ICZ, IIG, and thiophene blocks. The component located at 285.0 eV was assigned to C–S, C–N bonds of the three monomeric units. The N 1s peak shape (shown in Figure 2c) can be deconvoluted into two peaks, the peak at 399.5 eV can be attributed to C–N–C of the isoindigo block [32]. And the component at 400.0 eV is a typical position of a C–N–C bond in an ICZ unit. [33]. The relative filled areas of the two peaks in N 1s correspond to the feed ratios of IIG and ICZ units, respectively. The S 2p spectra illustrated in Figure 2d can be assigned to typical deconcvoluted S 2p3/2 (163.7 eV) and S 2p1/2 (164.8 eV) spin orbit splitting peaks, which was originated from C–S of the thiophene block. Figure 2e illustrated that two peaks of the O 1s profile can be deconvoluted, the peak located at 531.8 eV can be attributed to C=O unit of isoindigo block and the peak at 530.8 eV can be attributed to the metal oxide in ITO substrates. Similar results were obtained from the polymer PITID-1 (Figure S2 in Supplementary Materials). XPS curves of the two polymers showed that the random copolymers were successfully synthesized by incorporating the three monomers, indolo[3,2-b]carbzazole, isoindigo and thiophene units with different feed ratios.

### 3.3. Electrochemical Properties

In order to investigate the electrochemical properties of the materials, both of the random copolymers, dissolved fully in chloroform, were spray-coated homogeneously onto the conductive slide of the ITO substrates. The cyclic voltammetry tests of the copolymer films were conducted in the self-fabricated three-electrode system. The ITO glass substrates, Ag wire, and Pt wire were used as the working electrode, pseudo-reference electrode, and counter electrode, respectively. Cyclic voltammetry tests were carried out at the scan rate of 100 mV/s in a 0.2 M TBAPF_6_/ACN electrolyte solution between 0 and 1.8 V, accompanying the obvious redox peaks and instant color variation of the films. The variation can be originated from the emergence and disappearance of charge carriers, including polaron and bipolarons.

**Figure 3:**
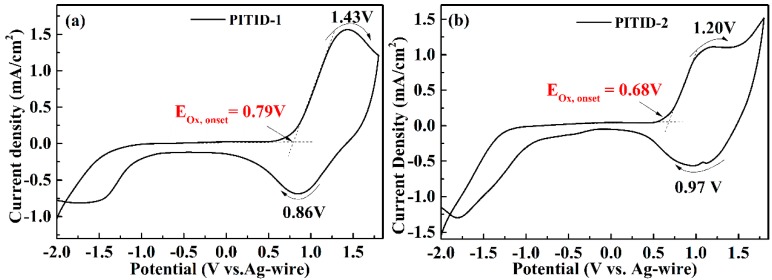
C–V curves of the copolymer films, PITID-1 (**a**) and PITID-2 (**b**), on the spray-coated indium tin oxide (ITO) substrates in 0.2 M TBAPF_6_/ACN electrolyte solution at a scan rate of 100 mV/s between −2 and 1.8 V.

As shown in Figure 3, the copolymers illustrated one obvious reversible redox peak at 1.43/0.86 V and 1.20/0.97 V for PITID-1 and PITID-2, respectively, during the p-type doping process. However, no obvious redox peaks were observed during the n-type doping scanning for the two polymers, it may originate from unstable anion radicals that can be easily disturbed by oxygen or water in the ambient conditions accompanying the transportation of the anions in the supporting electrolyte. For the polymer PITID-1 and PITID-2 (shown in Figure 3), the onset oxidation potentials (*E*_onset,ox_) were 0.79 V and 0.68 V, respectively. Compared with PITID-1, the lower values of *E*_onset,ox_ for PITID-2 may result from the smooth charge transfer between the donor and acceptor units due to the increased acceptor content (isoindigo unit).

### 3.4. Optical Behaviors of the Polymer Solutions and Films

**Figure 4:**
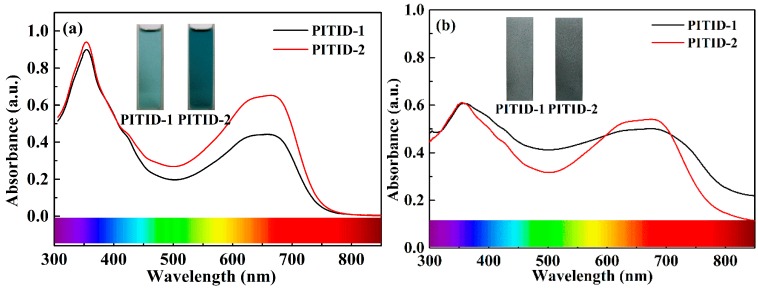
UV-vis absorption spectra of PITID-1 and PITID-2 in chloroform (**a**) and as thin films (**b**). The inserted pictures show the color of the polymers in different states.

The optical properties of the two different indolo[3,2-b]carbazole-based copolymers, PITID-1 and PITID-2, in the chloroform solutions and as thin films were studied using UV-vis-NIR absorption spectroscopy. The optical behaviors were shown in Figure 4 and the corresponding optical data was summarized in Table 2. As shown in Figure 4, both of the D–A type random copolymers possessed dual-absorption peaks with strong absorption intensity in visible region (380–720 nm) no matter in the form of solid or solution state, which is an unique characteristic of D–A type organic conjugated polymers. Figure 4a illustrated that the shorter-wavelength absorption band (λ_max,1,_ 300–480 nm), owing the same peak at 354 nm, originated from the localized π–π* transition of indolo[3,2-b]carbazole (ICZ) and thiophene units [34]. While the longer-wavelength absorption band (λ_max,2,_ 480–800 nm), centered at 658 nm, derived from intramolecular charge transfer (ICT) from ICZ and thiophene donor units to isoindigo acceptor unit. The valley of the curve at 500 nm can be observed. As the content of ICZ and thiophene decreased, the shorter-wavelength absorption band centered at 354 nm stayed unchanged, while the longer-wavelength absorption band centered at 658 nm red-shifted about 6 nm.

Compared with the spectra of polymer solutions in the visible region, the maximum absorption peaks of corresponding polymer films illustrated obvious red shift. The shorter-wavelength absorption peaks of both polymer films showed similar positions as in solution (at 355 and 353 nm for PITID-1 and PITID-2, respectively), whereas the longer-wavelength absorption peak shifted 6 nm up to 674 and 672 nm for PITID-1 and PITID-2, accompanying the decrease of relative intensity of two absorption bands. This behavior may be due to the aggregation effect of the polymer in the solid state. The onset absorption wavelength (λ_onset_) that had a negative correlation with the optical energy band gap (*E*_g_^opt^) was obtained as 795, 786 nm and the *E*_g_^opt^ values were calculated to be 1.56 and 1.58 eV for PITID-1 and PITID-2, respectively. From the inserted picture in Figure 4a,b, the solutions showed the colors of light cyan, dark cyan for PITID-1 and PITID-2, respectively. In the solid state, the color of the two polymers both illustrated dark gray that was not easy to identify by naked eyes.

### 3.5. Spectroelectrochemistry

Spectroelectrochemical properties of the spray-casted copolymer films PITID-X (X = 1, 2) that were deposited onto a conductive slide of ITO substrates were carried out to monitor the variation of absorption spectra ranging from 300 to 1600 nm as a function of different applied voltages.

**Figure 5:**
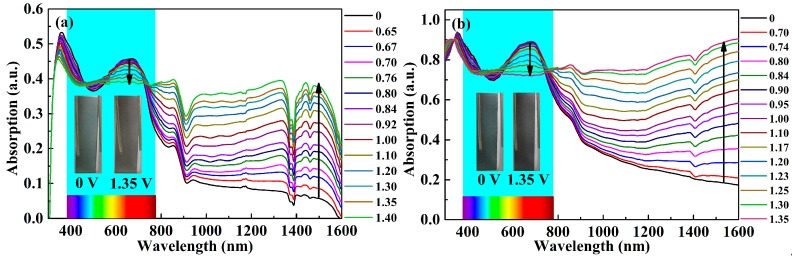
Two-dimensional (2D) spectroelectrochemistry profiles of (**a**) PITID-1 and (**b**) PITID-2 copolymer films on ITO-coated conductive slides in a 0.2 M TBAPF_6_/ACN electrolyte solution in different pulse voltages from 0 V to 1.35 V. The inserted photos show the color cyan in their neutral state (0 V, **left**) and the color gray in the oxidized state (1.35 V, **right**).

The photoelectric profiles of the two polymer films at different applied voltages ranging from 0 V (neutral state) to 1.35 V (oxidized states) were recorded in Figure 5. As shown in Figure 5a, the absorption curve of PITID-1 shows two absorption peaks, in the visible region at 355 and 674 nm in the neutral state. The first peak (λ_max,1_) centered at 355 nm with a wide absorption band from 300 to 500 nm and the second absorption peak (λ_max,2_), centered at 674 nm, showed a wider absorption band ranging from 500 to 960 nm, which can be originated from π–π* transitions and the ICT effect. The absorption valley at 500 nm allowed the transmittance of cyan light, which makes the PITID-1 film show the hue of cyan. However, it is almost transparent in the NIR region. The position of two absorption peaks may be related to the monomeric units and degree of polymerization in the copolymer chains, which was related to the intermolecular charge transition along the conjugated backbones. When the applied potential increased gradually from 0 V to 1.35 V, the absorption intensity of the two absorption peaks in the visible region reduced gradually and the absorption intensity in near infrared region, especially at the wavelength of 1500 nm, simultaneously greatly increased. It can be due to the decrease of π–π* transition electrons and the increase of polaron and bipolaron. The generation of polaron and bipolaron may facilitate the rearrangement of polymer structures, adjustment of HOMO and LUMO energy levels, and the value of energy gap. The color of the films changed from cyan (L* = 71.60, a* = −6.79, b* = 0.24) to gray (L* = 72.08, a* = −0.45, b* = −0.72) when the applied potentials gradually transferred from 0 to 1.35 V. Compared with the copolymer PITID-1, the spectra of PITID-2 was recorded at same applied potential, increasing from neutral state (0 V) to oxidized state (1.35 V). As shown in Figure 5b, the PITID-2 film also illustrated two similar intensity absorption bands. The position of absorption peaks at 353 nm and 672 nm can be obtained. As shown in Figure 5b, the film of PITID-2 illustrated obvious color transformation between the neutral and oxidized state, accompanying the color changing from cyan to gray. The color of the film PITID-2 changed from cyan (L* = 63.25, a* = 8.60, b* = −1.67) to gray (L* = 65.15, a* = 0.45, b* = 2.15) when the applied potentials transferred from the neutral state to the full-oxidized state.

### 3.6. Electrochromic Switching Study

Electrochromic switching study, including chronoamperometry and absorbance tests at specific wavelength under different redox states, is an important factor for evaluating the cycling stability and response time of the electrochromic materials. The parameter can also be used to calculate coloration efficiency of the sprayed films when the spectrophotometer was used in conjunction with electrochemical workstation in the three electrode systems. Electrochromic switching tests for the two copolymers films were studied via multi-potential steps for 300 s to monitor the change of optical contrast during the switching of the p-doping and p-dedoping process. At the same time, the current curves were recorded in the electrochemical workstation and the consumed charges and response time can be obtained during the single redox process.

**Figure 6:**
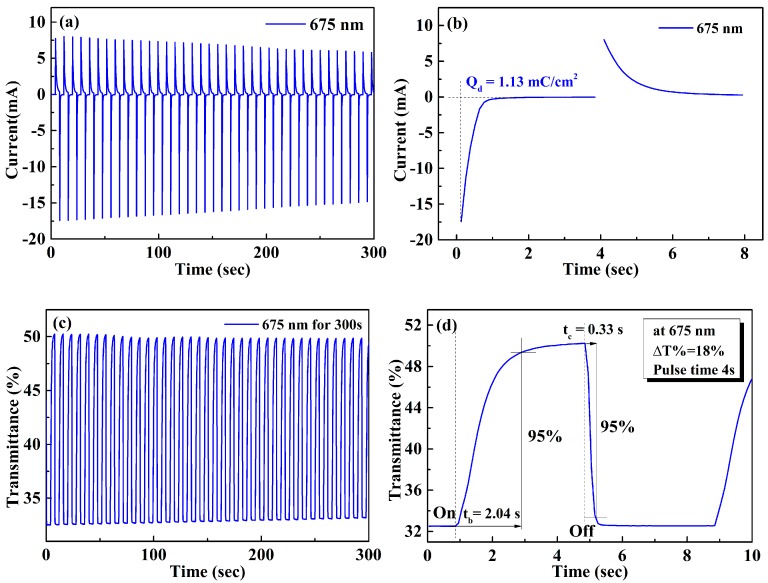
(**a**) Current–time switching curve of PITID-2 film between 0 and 1.35 V in a time interval of 4 s. (**b**) The second cycle of current–time curve. (**c**) Transmittance-time curve of PITID-2 last for 300 s at 675 nm (**d**) The bleaching time (*t*_b_) and the coloration time (*t*_c_) of PITID-2 at 675 nm.

For the copolymer PITID-2, the double absorption bands at 675 and 1600 nm were obtained from the spectroelectrochemical profiles with a wide absorption range at redox states. As shown in Figure 6a, the current curve was recorded and the current intensity decreased slightly in a 4 s switching interval after 300 s. We selected the second cycle to obtain the consumed charge density of 1.13 mC/cm^2^ at the wavelength of 675 nm, which were shown in Figure 6b. Figure 6c illustrates the optical transmittance changing percentage of PITID-2 film at 675 nm by switching the applied square-wave potential steps between 0 and 1.35 V for a 4 s time interval. The obtained transmittance contrast was 13% and then deduced slightly to 12% when the test time continued to 300 s. It is consistent with the reducing tendency of the current–time curve (shown in Figure 6a). The bleaching and coloration process were highlighted in Figure 6d. The bleaching process means the absorption percentage change from high value to low value. The coloration process means the transmittance percentage change from low value to high value. For the polymer PITID-2, the coloring response time that cannot be obtained instantly for the naked eyes for humans. Thus, it was calculated at 95% of a complete coloring and bleaching process to be 2.04 and 0.33 s in a 4 s time interval, respectively at 675 nm. Compared with PITID-2, the polymer PITID-1 illustrated similar values.

**Figure 7:**
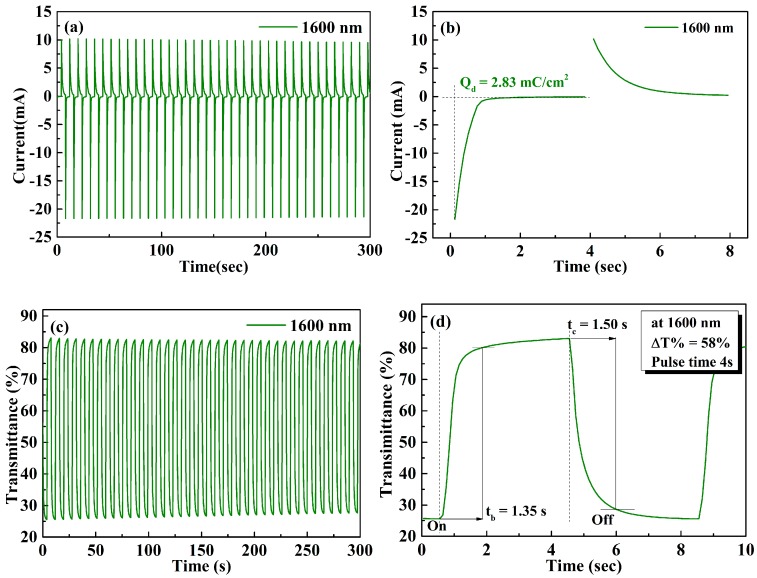
(**a**) Current–time switching curve of PITID-2 film between 0 and 1.35 V in a time interval of 4 s. (**b**) The second cycle of current–time curve. (**c**) Transmittance–time curve of PITID-2 last for 300 s at 675 nm. (**d**) The bleaching time (*t*_b_) and the coloration time (*t*_c_) of PITID-2 at 1600 nm.

When the testing absorption wavelength was changed to 1600 nm for PITID-2, the transmittance changing profiles (%∆*T*) were monitored as a function of time via UV-vis-NIR spectrophotometer and the switching current was recorded at the same time. Figure 7a depicted that the current underwent a slight loss in intensity during the whole doping and dedoping process between neutral and oxidized state in a time interval of 300 s. The consumed charge density was integrated to be 2.83 mC/cm^2^ from the second switching cycle (Figure 7b). Figure 7c illustrated that the transmittance changing percentage can stay from 58% to 56 % after 300 s, it was consistent with the current-time profile. As shown in Figure 7d, the 95% of complete bleaching and coloration times for PITID-2 were 1.35 and 1.50 s, respectively. The fast switching time and high optical contrast in the near infrared region of PITID-2 showed that the material owed potential application in the near infrared area. The kinetic properties of PITID-1 were slightly inferior to that of PITID-2 in several aspects including optical contrast, response time, and consumed charge. The kinetic figures of PITID-1 were shown in Figures S3 and S4 (in Supplementary Materials) in the Supporting Information. The corresponding data of PITID-1 were summarized in Table 3.

Coloration efficiency (CE; η) is an important parameter for evaluating the amount of consumed energy during the coloring or bleaching switching process. The value of η can be measured according to the following equation:

*H* = ∆*OD*/∆*Q*_d_ (1)

∆OD = lg(*T*_b_/*T*_c_) (2)

∆*Q*_d_ = *Q*/*A* (3)

where ∆*OD* represents the value of optical transmittance contrast between neutral/reduced and oxidized state and ∆*Q*_d_ represents the consumed charge during the process of injected or ejected electrons. According to the equation, the η values of PITID-1 were estimated to be 52.94 and 92.92 mC/cm^2^ at the wavelength of 670 and 1500 nm, respectively. The copolymer PITID-2 owed higher η values, which showed 171.52 and 153.08 mC/cm^2^ at 675 and 1500 nm, respectively. The results showed that the copolymer PITID-2 exhibited better coloration efficiency than PITID-1 and the material had better potential applications in electrochromic areas.

**Figure 8:**
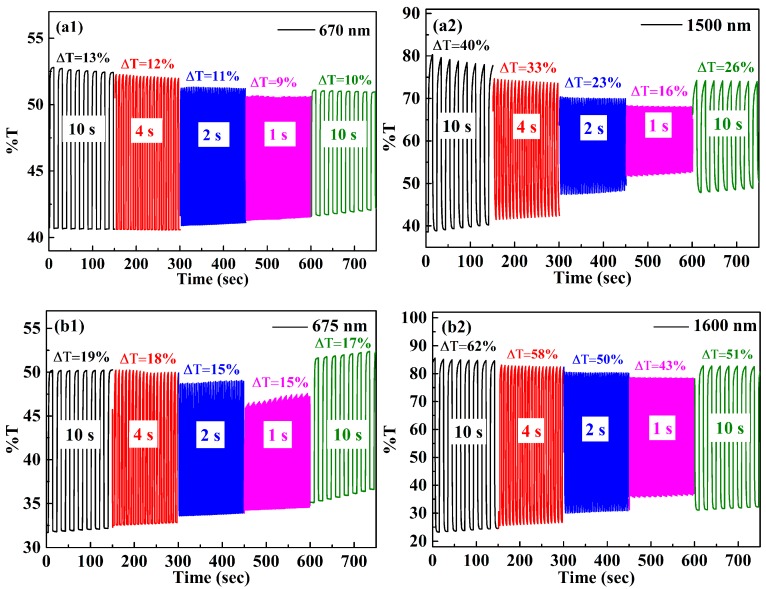
Square-wave potential step for PITID-1 (**a1**,**a2**) and PITID-2 (**b1**,**b2**) in a 0.2 M TBAPF_6_/ACN solution, by applying the potential from 0 to 1.35 V. (**a1**) 670 nm, (**a2**) 1500 nm, (**b1**) 670 nm, and (**b2**) 1600 nm. Switching time: 10 s step, 4 s step, 2 s step, 1s step, and back to 10 s, and 150 s for per step.

In practical applications of the electrochromic devices, the retention time at a certain voltage difference is dynamically variable, so it is very important to access the dependence of the optical contrasts on the retention times of the polymers during the dynamic switching. For this purpose, the interval times (at the neutral or oxidized state) were set at 10, 4, 2, 1, and back to 10 s, respectively, in the square wave potential step technique, and the optical contrasts were monitored. For PITID-1, the optical contrast at 670 nm were recorded as 12%, 11%, 7%, 5%, and 9%, respectively, at the interval time of 10, 4, 2, 1, and 10 s (Figure 8(a1)). As the time interval was reduced from 10 s to 1 s, the optical contrast was reduced by 31.2 %. At the wavelength of 1600 nm, the transmittance contrasts were 40%, 33%, 23%, 16%, and back to 26% at the interval time of 10, 4, 2, 1, and back to 10 s, respectively (Figure 8(a2)). There was a 30.6% loss in optical contrast as the switching time was decreased from 10 to 1 s. The relationship between the contrast differences and the time interval applied on the polymers was also investigated in the same way, and similar results were obtained for PITID-2 (Figure 8(b1,b2)). Based on the above data, it was found that optical contrasts have some degree dependence on the interval times, so it was necessary to estimate the response time at specific time intervals.

### 3.7. Colorimetric Analysis

In order to obtain the color and colorimetric characters of the two materials, the measurement with the CIE 1976 L*a*b* color space was used to record the instant color that cannot be distinguished by naked eyes during the variation of applied potentials. Here, L* represents the lightness of the color from black to white that can be transformed into values from 0 to 100. a* represents how much red (positive value) versus green (negative value) and b* represents how much yellow (positive value) versus blue (negative value). The positive values of a* and b* mean red and yellow, respectively, or vice versa.

**Figure 9:**
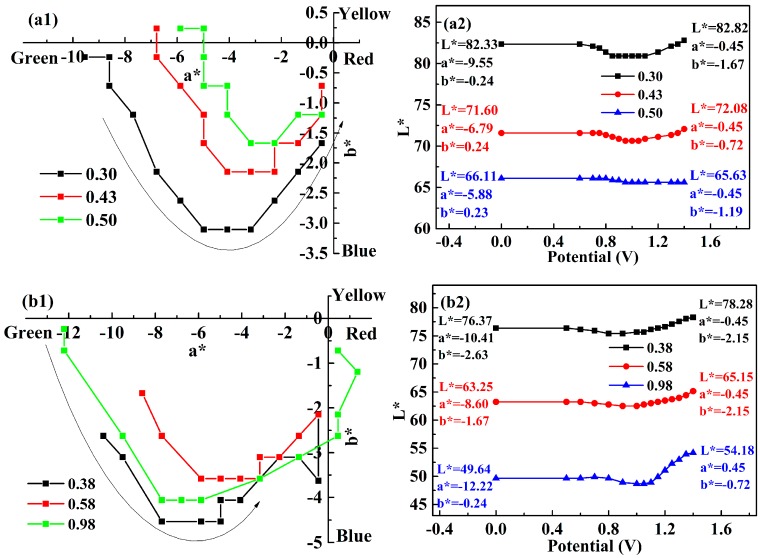
CIE L*a*b* color coordinates for the copolymers PITID-1 (**a**) and PITID-2 (**b**) films during p-doping processing from neutral state to oxidized states with three measurable thicknesses.

As can be seen from Figure 9(a1,b1), the initial a*, b* values of polymer PITID-1 and polymer PITID-2 located at the negative abscissa, which stood for cyan color in the neutral state. As seen in Figure 9(a1), the color change trajectories of PITID-1 films with different thickness are identical, i.e., from gray to a blue-green color, and then to a gray-blue color, finally turning to a gray color. Polymer PITID-2 had the very similar color changes during the step-by-step p-doping process, as shown in Figure 9(b1). The color space coordinate clearly presented the color changing process under different potentials. The L* values for polymer films (PITID-1) with different thickness are also presented in Figure 9(a2), which have significant changes at 0.6 V and return to neutral light at 1.35 V. Similar with PITID-1, the L* values for the PITID-2 polymer with different thickness decreased slightly during the p-doping process and back to neutral light at 1.35 V (Figure 9(b2)). Apart from the similarity in the neutral state color and the oxidized state color, polymer PITID-2 experienced a similar color changing process with that of PITID-1. The polymer eventually changed from a cyan color to a gray color as the polymer switched from the neutral state to the fully oxidized state. In addition, the L* values for the two polymers slightly increased as the potential difference increased from 0 to 1.35 V.

### 3.8. Open Circuit Memory Experiments of the Two Polymers

**Figure 10:**
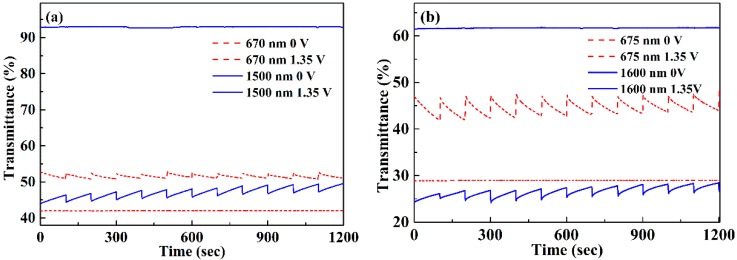
Open circuit stability of the sprayed films at 670 and 1500 nm for copolymer PITID-1 (**a**) and the film at 675 and 1600 nm for copolymer PITID-2 (**b**). The pulse voltage was applied for 1 s for every interval time of 120 s to recover the initial transmittance at constant neutral or oxidized state. Straight line: neutral state, Jagged line: oxidized state. Dotted line: 670/675 nm, Solid line: 1500/1600 nm.

Open circuit memory property is an important factor for electrochromic materials or devices, the long residence time can reduce the energy consumption during the switching process of electrochromic devices. The open circuit memory curves of the copolymers films that were applied at constant potential (0 or 1.35 V), were recorded at the wavelength of 670/1500 nm and 675/1600 nm for 1 s for every 120 s time interval for PITID-1 and PITID-2, respectively. As shown in Figure 10a, the film of PITID-1 kept an unchangeable transmittance at 670 nm when the film was in the neutral state. However, the profile show jagged curve as the polymer was kept in the oxidized state by intermittent potential at 1.35 V for 1200 s, unstable but recoverable absorption retention characteristics. Similar variation tendency can be obtained for PITID-2 in Figure 10b. The results show that the two materials show good optical memory at the neutral state, and the memory in the oxidized state could be compensated by the pulse applied voltages.

### 3.9. Thermal Properties

**Figure 11:**
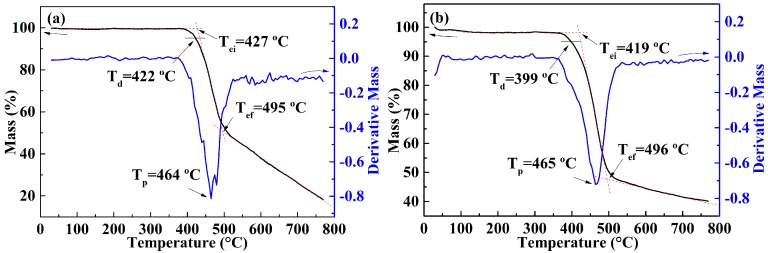
Thermogravimetric (TG) and differential thermogravimetric (DTG) curves for PITID-1 (**a**) and PITID-2 (**b**).

The thermal behaviors and stabilities of the conjugated materials PITID-1 and PITID-2 were studied via thermogravimetric analysis (TG; TGA) and differential thermogravimetric (DTG) methods. Samples were heated at the heating rate of 10 °C/min from room temperature to 750 °C. Several physical parameters are obtained from TG and DTG curves (Figure 11). The initial decomposition temperature (*T*_d_) refers to the point at which a 5% weight loss of the material occurs. The prestissimo decomposition temperature (*T*_p_) represents the inflection point that can be drawn from the peak value of DTG curve. The extrapolated finish temperature (*T*_ef_) is the temperature at the intersection point of the tangent line of the mid-section and the tail section of the TG curve. The *T*_ei_ temperature refers to the intersection of the tangent of the baseline and the tangent line of the mid-section curve of the TG curve. The % char refers to the percentage of the residual carbon ash to the quality of the initial samples at the final temperature (750 °C in this case). The data were summarized in Table 4. TGA plots showed that both copolymers exhibited good thermal stabilities in N_2_ atmosphere, with the T_d_ temperatures were observed at 422 °C (PITID-1) and 399 °C (PITID-2). The high decomposition temperature may benefit from the rigid conjugated groups with long alkyl side chains. PITID-1 has a *T*_p_ temperature of 464 °C, a *T*_ei_ temperature of 427 °C, a *T*_ef_ temperature of 495 °C. The % char of the polymers were 20% for PITID-1 and 41% for PITID-2. The polymer PITID-2 possessed similar thermal parameters to that of PITID-1. The materials can endure over 400 °C in test condition without decomposition and can meet the requirements on environmental temperature for various electrochromic devices.

## 4. Conclusions

The two random polymers PITID-X (X = 1, 2) based on indolo[3,2-b]carbazole, isoindigo and thiophene units were successfully synthesized via Stille cross-coupling polymerization with different raw material feed ratios. It was feasible to obtain the color of the materials with two characteristic absorption peaks by adopting the strategy of changing variable donor-to-acceptor ratio of the copolymers. ^1^H NMR, IR, XPS measurements were carried out to confirm the structural units of the copolymer chains. TGA curves showed that the polymers owed stable heat tolerance property over 400 °C. The CV curves of the polymers showed one pair of redox peaks, which suggested the obvious reversible p-type doping/dedoping ability. The UV-vis-NIR profiles of the films illustrated that the spray-coated films showed more obvious red shift than that of corresponding polymer solutions. The results of faster response time, higher optical contrast in NIR, and better kinetic stability showed that the electrochromic properties of PITID-2 were superior to that of PITID-1. For PITID-2, it had a color change from cyan (neutral state) to gray (oxidized state), showed good electrochromic property due to low optical band gap of 1.54 eV. The materials also illustrated fast bleaching/coloring response time of 2.04/0.33 s and 1.50/3.37 s, good coloration efficiency of 171.52 and 153.08 cm^2^ C^−1^ and satisfactory optical contrast of 18% and 58% at wavelengths of 675 and 1600 nm, respectively.

## Figures and Tables

**Table 1:** Electrochemical data and molecular weights of the PITID-1 and PITID-2.

Copolymer	*M*n	*M*w	PDI	Yield	*E*_onset,ox_	Redox Peak
	/kDa	/kDa			/V	/V
PITID-1	20.1	27.5	1.37	74	0.79	1.43/0.86
PITID-2	17.7	23.7	1.34	73	0.68	1.20/0.97

Estimated from CV data vs. Ag wire, in which Eonset,ox refers to the oxidation onset potentials of the polymers (vs. Ag wire electrode).

**Table 2:** Summary of optical and electrochemical properties of copolymers PITID-X (X = 1, 2) in solution and as film state.

Compound	In Solution		In Film State		^a^ *E*_g_^opt^	^b^ *E*_onset_	^c^ HOMO (eV)	^d^ LUMO (eV)
	λ_max_ (nm)	λ_onset_ (nm)	λ_max_(nm)	λ_onset_ (nm)	(eV)	(V)		
PTDDB-1	354; 658	744	355; 674	795	1.56	0.79	−5.04	−3.48
PTDDB-2	354; 664	748	353; 672	786	1.58	0.68	−4.93	−3.35

^a^*E*_g_^opt^ is calculated following the equation: *E*_g_^opt^ = 1240/λ_onset_^film^; ^b^ *E*_onset_ data is obtained from CV curve. The oxidation peak potential for Fc^+^/Fc is calculated to be 0.55 V vs. Ag wire. ^c^ *E*_HOMO_ = −[4.8 + (*E*_onset,ox_ − 0.55)] eV; ^d^ *E*_LUMO_ = *E*_HOMO_ + *E*_g_^opt^.

**Table 3:** Electrochromic performance of PITID-1 and PITID-2 polymer films.

Polymer	λ_max_	%∆*T*	^a^ Response Time		^b^ ∆*OD*	^c^ ∆*Q*_d_	^d^ *CE*
	nm		*t*_c_(s)	*t*_b_(s)		(mC cm^−2^)	cm^2^ C^−1^
PITID-1	670	12	2.89	0.39	0.09	1.08	52.94
	1500	33	2.36	2.23	0.25	2.71	92.92
PITID-2	675	18	2.04	0.33	0.19	1.13	171.52
	1600	58	1.5	1.35	0.51	3.37	153.08

^a^ the response time is calculated at 95% of the full transmittance change. ^b^ △OD represents the value of optical transmittance contrast between neutral/reduced and oxidized state. ^c^ △Q_d_ represents the consumed charge during the process of injected or ejected electrons. ^d^ Coloration efficiencies (CE; η) are obtained from Equation (1).

**Table 4:** TG and DTG data for PITID-1 and PITID-2.

Compound	*T*_ei_ (°C)	*T*_ef_ (°C)	*T*_p_ (°C)	*T*_d_ (°C)	% char
PITID-1	427	495	464	422	20
PITID-2	419	496	465	399	41

## Supplementary Materials

The following are available online at https://www.mdpi.com/2073-4360/11/10/1626/s1. Figure S1: ^1^H NMR spectra of the copolymers PITID-1 (a) and PITID-2 (b). Figure S2: High-resolution XPS spectra of the copolymer PITID-1; (a) survey scan; (b) C 1s; (c) N 1s; (d) S 2p; (e) O 1s, The raw and fitted curves were recorded in solid and dotted lines, respectively. Figure S3: (a) Current–time switching curve of PITID-1 film between 0 and 1.35 V in a time interval of 4 s; (b) The second cycle of current–time curve; (c) Transmittance–time curve of PITID-1 last for 300 s at 670 nm; (d) The bleaching time (t_b_) and the coloration time (t_c_) of PITID-1 at 670 nm. Figure S4: (a) Current–time switching curve of PITID-1 film between 0 and 1.35 V in a time interval of 4 s; (b) The second cycle of current–time curve; (c) Transmittance–time curve of PITID-1 last for 300 s at 1500 nm; (d) The bleaching time (t_b_) and the coloration time (t_c_) of PITID-1 at 1500 nm.
