# Supplementary material for: Design and Characterization of New D–A Type Electrochromic Conjugated Copolymers Based on Indolo[3,2-b]Carbazole, Isoindigo and Thiophene Units
Source: Polymers (Basel). 2019 Oct 8;11(10):1626. doi: 10.3390/polym11101626 (PMC6836008; doi:10.3390/polym11101626)
Supplement: Supplementary file 1 [file polymers-11-01626-s001.pdf]

polymers

MDPI

Supporting Information

Design and Characterization of New D-A Type Electrochromic Conjugated Copolymers Based on Indolo[3,2-b]Carbazole, Isoindigo and Thiophene Units

Yuling Zhang [1,2], Shuang Chen [1,*], Yan Zhang [2], Hongmei Du [2] and Jinsheng Zhao [2,3,*]

[1] State Key Laboratory of Heavy Oil Processing, College of Chemical Engineering, China University of Petroleum (East China), QingDao 266580, China; 18866264190@163.com (Y.L.Z.)

[2] Shandong Key Laboratory of Chemical Energy Storage and Novel Cell Technology, Liaocheng University, Liaocheng 252059, China; zy@lcu.edu.cn (Y.Z.); duhongmei@lcu.edu.cn (H.D.)

[3] College of Chemistry and Chemical Engineering, Liaocheng University, Liaocheng, 252059, China

* Correspondence: chsh1030@163.com (S.C.); j.s.zhao@163.com (J.Z.)

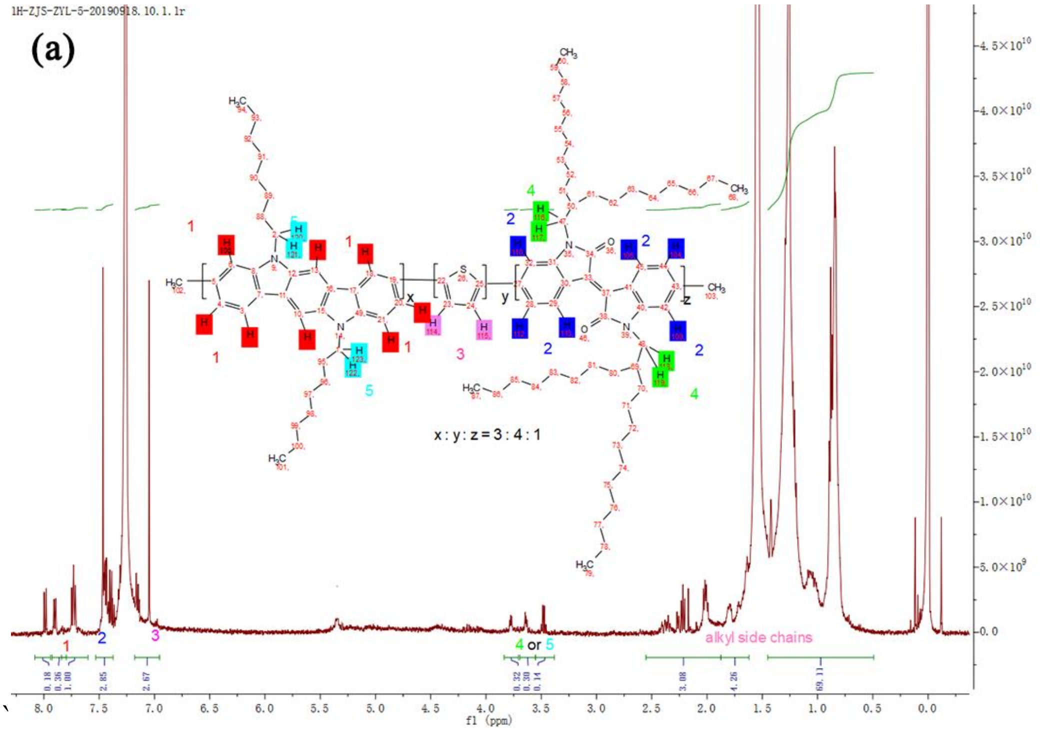

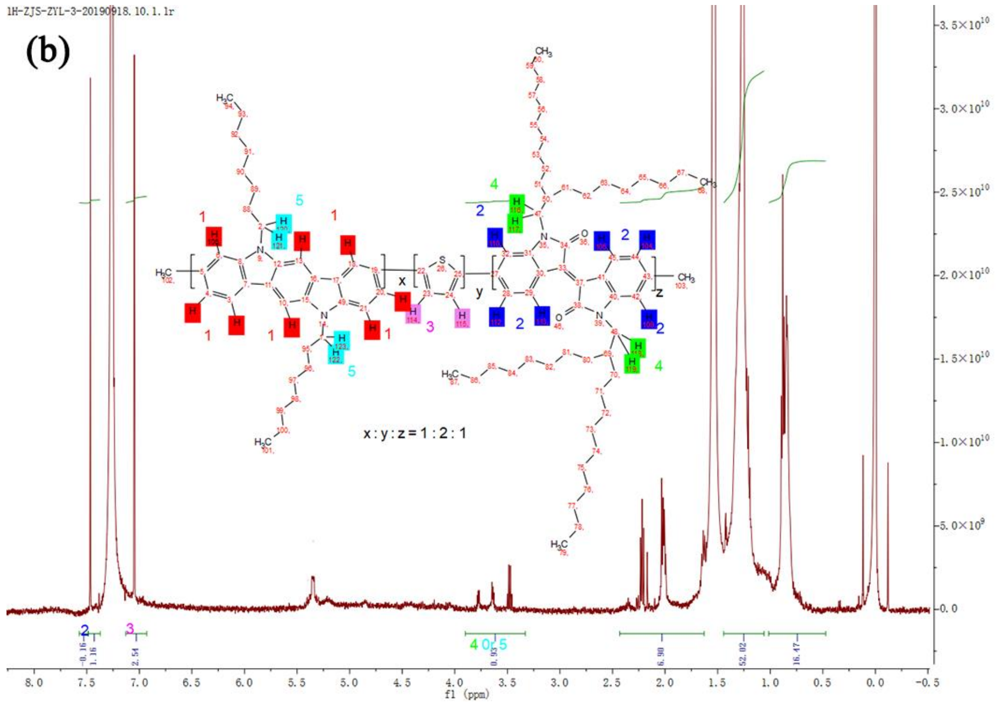

Figure S1. ^{1}H NMR spectra of the copolymers PITID-1 (**a**) and PITID-2 (**b**).

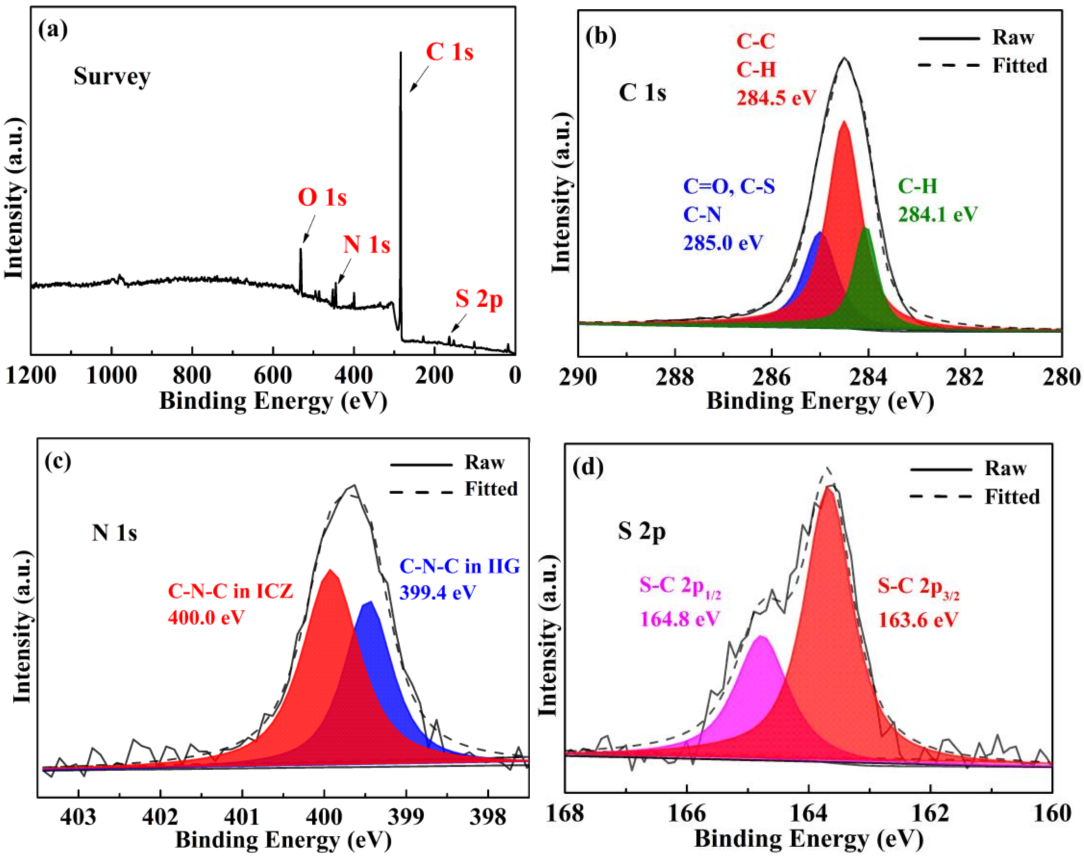

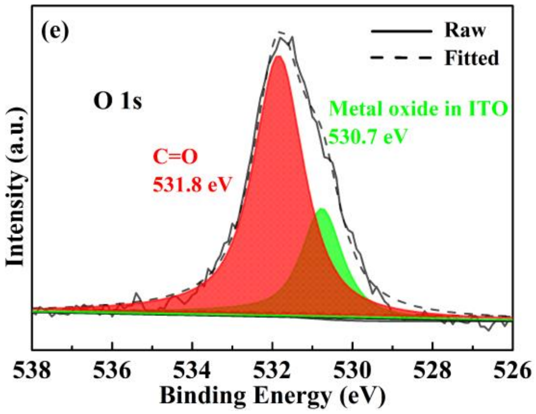

Figure S2. High-resolution XPS spectra of the copolymer PITID-1; (**a**) survey scan; (**b**) C 1s; (**c**) N 1s; (**d**) S 2p; (**e**) O 1s. The raw and fitted curves were recorded in solid and dotted lines, respectively.

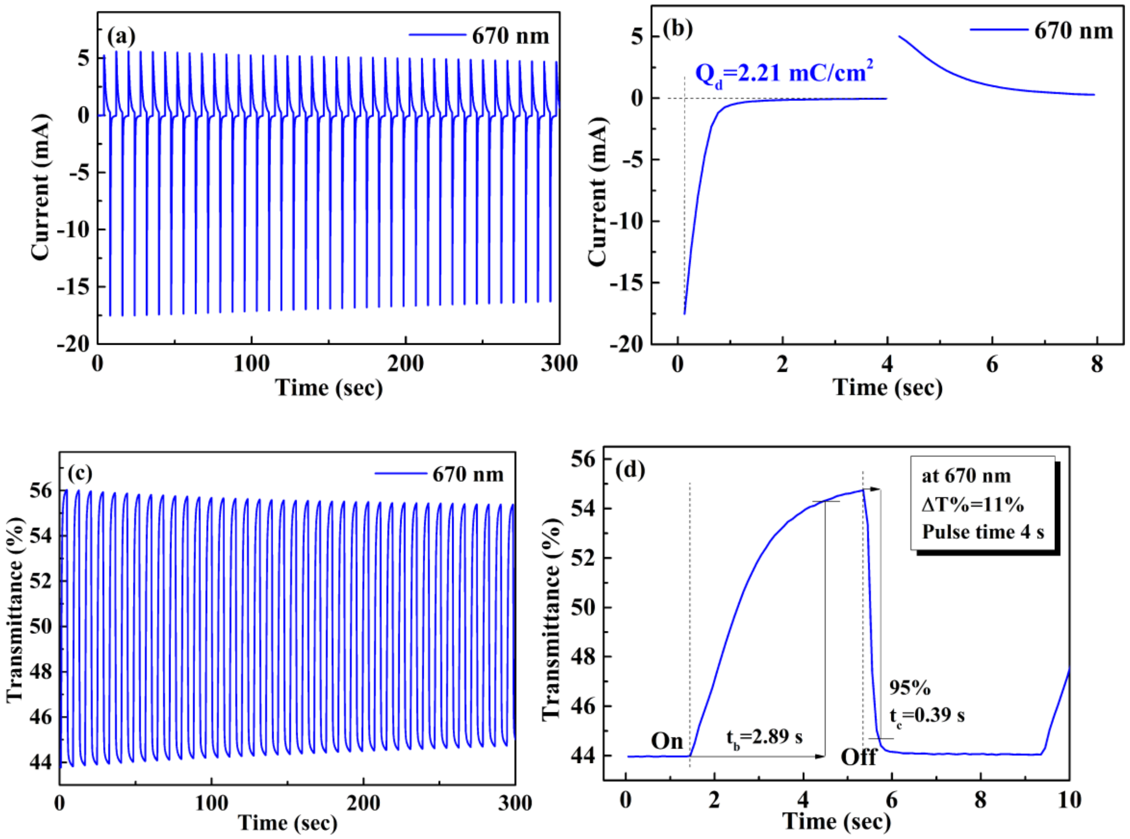

Figure S3. (**a**) Current–time switching curve of PITID-1 film between 0 and 1.35 V in a time interval of 4 s. (**b**) The second cycle of current–time curve. (**c**) Transmittance–time curve of PITID-1 last for 300 s at 670 nm. (**d**) The bleaching time (t_b) and the coloration time (t_c) of PITID-1 at 670 nm.

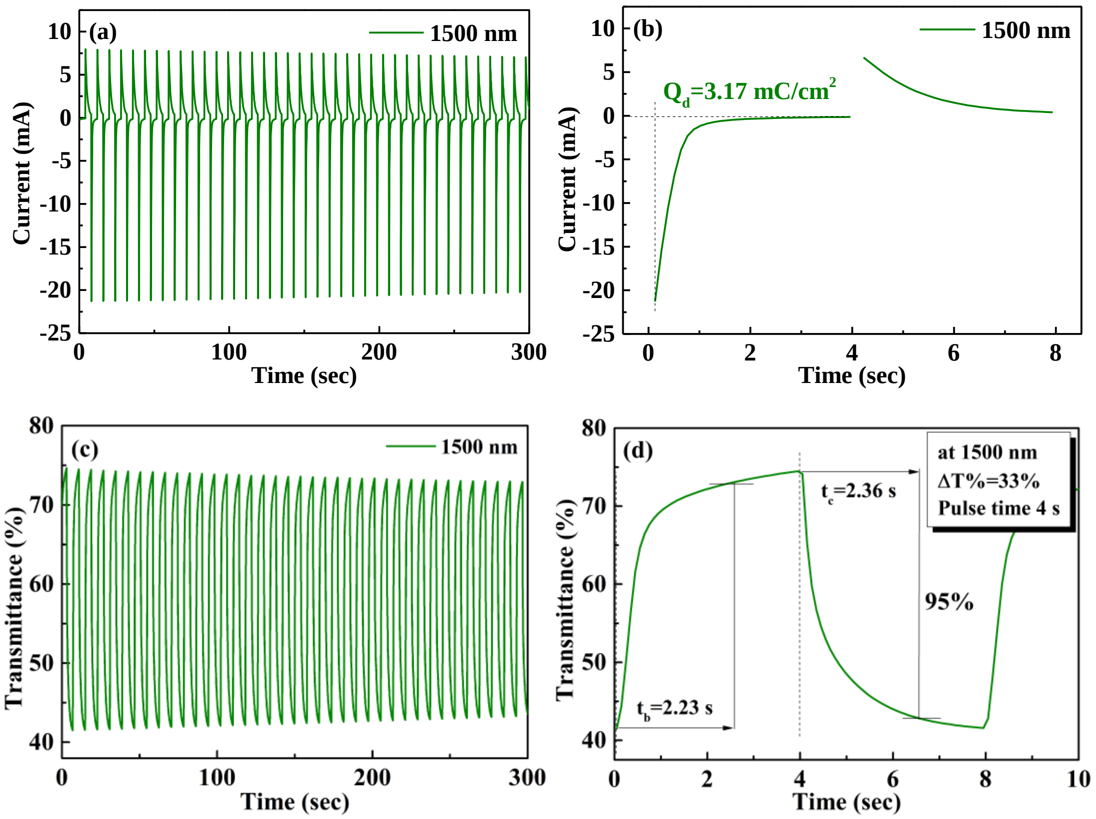

Figure S4. (**a**) Current–time switching curve of PITID-1 film between 0 and 1.35 V in a time interval of 4 s. (**b**) The second cycle of current–time curve. (**c**) Transmittance–time curve of PITID-1 last for 300 s at 1500 nm. (**d**) The bleaching time (t_b) and the coloration time (t_c) of PITID-1 at 1500 nm.
